# The Effect of Perceived Effort on Reward Valuation: Taking the Reward Positivity (RewP) to Dissonance Theory

**DOI:** 10.3389/fnhum.2020.00157

**Published:** 2020-05-14

**Authors:** Eddie Harmon-Jones, Daniel Clarke, Katharina Paul, Cindy Harmon-Jones

**Affiliations:** ^1^School of Psychology, The University of New South Wales, Sydney, NSW, Australia; ^2^Department of Experimental, Clinical, and Health Psychology, Ghent University, Ghent, Belgium

**Keywords:** effort, reward, cognitive dissonance, reward positivity, event-related potentials

## Abstract

The present research was designed to test whether the subjective experience of more effort related to more reward valuation as measured by a neural response. This prediction was derived from the theory of cognitive dissonance and its effort justification paradigm. Young adult participants (*n* = 82) engaged in multiple trails of a low or high effort task that resulted in a loss or reward on each trial. Neural responses to the reward (loss) cue were measured using EEG so that the event-related potential known as the Reward Positivity (RewP) could be assessed. Results revealed no significant differences between low and high effort conditions on the RewP. However, within the high effort condition, a more subjective experience of effort was associated with a larger RewP. This research extends past research on the effort justification paradigm of cognitive dissonance theory by suggesting that effort justification is associated with an implicit measure of reward valuation. It, therefore, challenges recent perspectives on dissonance processes that posit that these evaluative changes should only occur on explicit but not implicit measures.

## Introduction

According to cognitive dissonance theory and its research (Festinger, 1957; Levy et al., 2018), the more effort one exerts, the more valuable one perceives the reward associated with that effort. This effect has been referred to as *effort justification*. Cognitive dissonance theory proposes that elevating the attractiveness of a reward following the exertion of unpleasant effort is a motivated process that serves to reduce psychological inconsistency regarding having engaged in the effort (Festinger, 1957; Harmon-Jones and Mills, 1999, 2019).

In one classic experiment, Aronson and Mills (1959) had female participants undergo an initiation to gain access to a group discussion. The initiation was designed to be of low or high effort (and another condition simply listened to and rated the group discussion). The low effort initiation involved reading sexually-oriented material that was not very embarrassing, whereas the high effort initiation involved reading sexually-oriented material that was very embarrassing. After undergoing the initiation, participants listened to a group discussion about sexual behavior in animals. Results revealed that participants who underwent the more embarrassing (effortful) initiation evaluated the group more positively than participants who underwent the mildly embarrassing initiation. This classic experiment used the amount of embarrassment as the manipulation of effort. Other dissonance experiments have manipulated effort in a variety of ways, including the difficulty of cognitive tasks (Axsom and Cooper, 1985; Harmon-Jones et al., 2015b).

According to cognitive dissonance theory, individuals value that for which they have suffered to reduce the dissonance from engaging in the effort. The cognition, “I underwent embarrassment to gain admission to this group,” is in contradiction with the cognition, “I would prefer not to undergo embarrassment.” The dissonance from this discrepancy can be reduced by justifying the effort by adding a cognition that is consonant with the former cognition, “The group is interesting and desirable.” The variables of critical importance in dissonance theory are psychological or subjective (e.g., the perception of effort).

Past dissonance theory research on effort’s influence on reward valuation has measured subjective evaluations of rewards. Indeed, some perspectives posit that dissonance reduction should not influence implicit measures such as neural responses (Gawronski and Brannon, 2019). The present research sought to extend past research by testing whether the effort would increase reward valuation when it was measured using a neural response. One such response that has been studied extensively is an event-related potential referred to as the Reward Positivity (RewP). This response has also been referred to as the feedback negativity (Yeung and Sanfey, 2004) and feedback-related negativity (Gehring and Willoughby, 2002). This response is often examined by computing the difference between the event-related potentials (ERP) to gains and losses approximately 300 ms following performance-related feedback. Much research has now suggested that this ERP difference wave is primarily driven by responses to receipt of gain and not loss cues (for reviews see Proudfit, 2015; Krigolson, 2018). However, this position is not completely accepted (Yaple et al., 2018). The RewP is associated with the likelihood and magnitude of reward (Sambrook and Goslin, 2015). The RewP is associated with activity in a “reward” neural circuit involving the ventral striatum, medial prefrontal cortex, anterior cingulate cortex, orbital frontal cortex, amygdala, and caudate (Carlson et al., 2011, 2015; Becker et al., 2014).

Larger RewPs are associated with greater extraversion (Cooper et al., 2014), trait approach motivation (Lange et al., 2012), trait reward responsiveness (Bress and Hajcak, 2013), and greater state and trait anger (Angus et al., 2015; Tsypes et al., 2019). In contrast, smaller RewPs are associated with greater state-induced sadness (Foti and Hajcak, 2010) and depressive symptoms (Foti and Hajcak, 2009). Moreover, a manipulated increase in perceived control over obtaining rewards causes an increase in the RewP (Mühlberger et al., 2017). These results suggest that the RewP is associated with higher approach motivation and likely to be increased with effort, as effort is often associated with approach motivation.

The present study was designed to test whether increased effort would be associated with increased reward valuation as predicted by dissonance theory. We wanted to assess whether we could observe this predicted effect using the RewP as a dependent variable, as most past dissonance/effort justification studies used self-reported attitudes toward the reward as the dependent variable. We predicted that individuals who exert high effort to obtain a reward should have a larger RewP than individuals who exert low effort to obtain the same reward. Also, because dissonance theory’s predictions are based on the subjective variable of perceived effort, we tested whether individuals who perceive themselves to have engaged in more effort have a larger RewP.

The present research was designed to test the prediction derived from cognitive dissonance theory that effort should increase reward valuation. However, other perspectives have suggested that effort should decrease reward valuation (e.g., Inzlicht et al., 2018). To date, no clear resolution of these competing predictions has been proposed, but dissonance theory and its research have suggested that effort should increase reward valuation primarily when individuals believe the reward is contingent on the effort (i.e., when they perceive control over the outcome; Gerard and Mathewson, 1966). Based on this past research, the present research gave participants perceived control over the outcome.

## Materials and Methods

### Participants

Participants were 90 right-handed individuals who participated in exchange for course credit in their first-year psychology class or monetary compensation ($40 in Australian dollars; AUD). Of these, eight participants were excluded: one for poor performance on the number judgment task, three because they stated they did not believe that receiving a reward was dependent on their performance, and four because they had fewer than 20 artifact-free trials for either reward or loss feedback, leaving 82 (46 male, *M* = 20.74, *SD* = 3.64) for the analyses. Reported ethnicities were White (31.7%), Northeast Asian (34.1%), Southeast Asian (28.0%), Southcentral Asian (9.8%), Middle Eastern (4.9%), and other (3.7%). The UNSW Human Research Ethics Advisory Panel for Psychology HREAP-C approved the study, protocol #2948.

### Design

The experiment was a two condition (low effort, high effort) between-subjects design. Effort was manipulated by assigning participants to an easy (low effort) or difficult (high effort) version of a number judgment task.

### Procedure

Participants were seated at a computer in an individual testing room where they provided informed consent. A jar containing money was visible on the table to ensure that participants believed that they would receive money after the experiment.

After the researcher attached the EEG sensors to the participant, he explained that they would gain money during the task and instructed them to read the instructions presented on the computer screen. He then left and closed the door to the participant’s room and started the experiment. The researcher remained blind to condition throughout the experiment^1^.

Participants completed demographic questions (see above) and filler personality questionnaires (these measures were not analyzed because they were not relevant to the hypothesis; thus, they are not reported). The completion of these questionnaires was included so that all participants would be in a similar, neutral mindset. Participants then completed the number judgment task while EEG was measured. Then, they completed self-report measures. After the experiment, participants were debriefed and given their monetary reward (which was approximately AUD 7.00).

### Materials and Measures

#### Instructions and Practice for Number Judgment Task

Before beginning the task, participants were informed that whether they received the reward for each block would depend on their speed, accuracy, and other variables. However, during the task, reward feedback was presented almost randomly so that participants would receive an equal number of rewards and losses. The only performance criterion during the actual task was whether the participant failed to respond to any trials within a block during the 1,500 ms when the numeral was shown; in such cases, they received loss feedback.

Practice blocks familiarized participants with the procedure. Participants first completed a parity or magnitude block (low effort) followed by a switching block (high effort), with no reward feedback (see below for details of parity, magnitude, and switching blocks). Then, if they were assigned to the low effort condition, they completed a parity and a magnitude practice block, whereas if they were assigned to the high effort condition, they completed two switching practice blocks. If participants were correct on less than 80% of trials, they completed two more practice blocks.

#### Number Judgment Task

The task, adapted from one used by Botvinick et al. (2009), consisted of individual numbers being shown in yellow or blue font; the numbers ranged from 1 to 9, excluding 5. If the number was in yellow font, participants were instructed to judge whether it was greater or less than 5 (magnitude judgment). If the number was in blue font, participants were instructed to judge whether it was odd or even (parity judgment). The numbers were presented one at a time and shown in blocks of 10. Each number was presented until participants made a response using one of the response keys or for a maximum of 1,500 ms. If participants did not respond within 1,500 ms, an incorrect response was registered for that number. A 500 ms inter stimulus interval occurred between the presentation of each number.

Each block of 10 number presentations was preceded by a cue that indicated the type of block. In the *low effort condition*, a solid blue or yellow circle cue was presented (2,000 ms). A blue circle indicated that all numbers would be blue and thus all judgments would be based on parity (odd, even). A yellow circle indicated that all numbers would be yellow and thus all judgments would be based on magnitude (greater or less than 5). In the *high effort condition*, a half-blue, half-yellow circle cue was presented (2,000 ms), and this indicated that participants would have to alternate between making parity and magnitude judgments. Because this block required task switching, it was more difficult and effortful than the parity or magnitude block.

**Figure 1:**
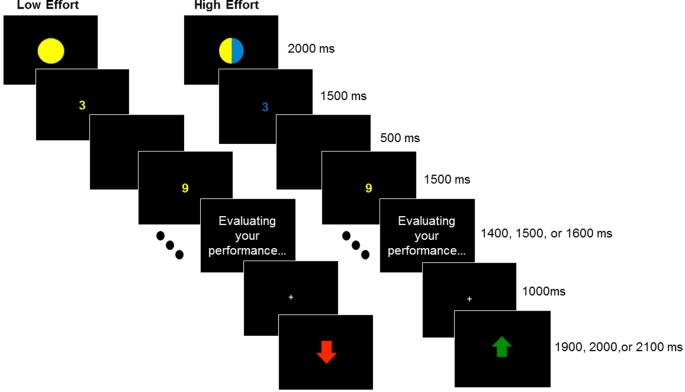
Low1 effort (left) and high effort (right) blocks for the number judgment task. Each block included 10 numerals and ended with either reward or loss feedback.

Following each block of 10 trials, participants viewed the message, “Evaluating your performance …” for 1,400, 1,500, or 1,600 ms. This was followed by a fixation cross for 1,000 ms, followed by the green up-arrow reward cue (indicating that they would receive $0.40) or red down-arrow loss cue (indicating that they would lose $0.20) for 1,000 ms. Then, 1,900, 2,000, or 2,100 ms interblock intervals occurred before the next block. These reward and loss amounts of money were chosen because they proved effective in evoking psychophysiological responses in previous research (Angus and Harmon-Jones, 2019). Moreover, because losses are approximately twice as subjectively impactful as gains, rewards were twice as large in magnitude as losses. Participants completed 80 blocks of the number judgment task. See Figure 1.

#### Self-report Measures

After the number judgment task, participants were asked, “How difficult did you find the task?” Responses were on a scale from 1 = not very difficult to 7 = very difficult. We chose to use a simple, direct, face-valid measure of self-reported task difficulty, as has been used in previous research (Wright et al., 1990, 1995). This perceived task difficulty measure was used to assess perceived effort. We used self-reported task difficulty to measure perceived effort because: (1) effort is defined as “an attempt to do something that is difficult or that involves hard work” (Effort, 2009); (2) we suspected that self-reported effort might be contaminated by self-presentational concerns, as when participants might report engaging in high levels of effort on most tasks because they want to appear as though they are doing what is expected of them; and (3) our experience using self-reported effort in other lines of research taught us that it was an insensitive index (Wright et al., 1990, 1995).

### EEG Recording

EEG was recorded using a BioSemi Active Two AD-box with 24-bit resolution and was received using Actiview version 7.07 software, with a sampling rate of 512 Hz and a 50 Hz notch filter.

BioSemi 32 set Ag-AgCl Pin-Type Active electrodes were used (model number P32-1020-32Acms), applied using the 10-20 system. A head cap made from an elastic fabric with 32 electrode holders was used to mount the electrodes; eight additional BioSemi Ag-AgCl FLAT Active electrodes were used as external electrodes. Electrode collars were used to attach the external electrodes. To act as reference electrodes, two external electrodes were placed on the right and left mastoids. To measure eye movements, four external electrodes were placed above and below the right eye in line with the pupil, and on the outer side of the right and left eye in line with the pupil. To measure electrocardiography, two external electrodes were placed on the right and left side of the chest (this measure was not relevant to the present hypothesis and was therefore not analyzed). Parker SignaGel was used for electroconductivity between the skin and the electrodes.

### EEG Data Processing and Analysis

EEG data were analyzed off-line using Brain Vision Analyser 2.1. Poor performance trials (<80% correct) were excluded from the analysis for each participant. This was done to address interpretational issues associated with the meaning of ERP responses following poor performance, including the possibility that participants did not exert effort or were not paying attention and thus did not attend to the feedback stimuli. Electrode sites FP1, FP2, T7, and T8 were also excluded for all participants as these sites contained muscle artifacts for the majority of participants and are not relevant to the RewP.

The remaining continuous raw data for all channels were re-referenced to the average of the mastoid sites and filtered using a band-pass filter with 0.1 and 30 Hz cut-offs (Bress and Hajcak, 2013). The data were then segmented into 1,000 ms segments, starting 200 ms before the feedback presentation (baseline) and ending 800 ms after the onset of the feedback (Bress and Hajcak, 2013). Eye movement was corrected using the method of Gratton et al. (1983) and a baseline correction was applied to account for any inter-trial differences. Artifacts were removed with an automated procedure. The following criteria were used to determine artifacts:

(1) maximal allowed absolute difference of 300 μV across the entire interval (−200 to 800 ms); (2) maximal allowed voltage step of 50 μV (the maximal difference in voltage between two data points); and (3) a minimum allowed activity of 0.50 μV in a 100 ms interval (Bress and Hajcak, 2013).

The segments were then separated by feedback type and condition (e.g., high effort, reward; high effort, loss) and the data were averaged for each of these groups. The mean values for electrodes Fz and Cz from 220 ms to 320 ms were exported and used for the statistical analysis. These sites are implicated in the RewP (Proudfit, 2015). This is in line with previous ERP research which analyses a 100 ms window around the peak of the difference wave (Proudfit, 2015).

## Results

### Task Difficulty Manipulation Check

To test whether the difficult task was perceived as more difficult than the easy task, participants’ self-reported task difficulty was compared between conditions. Participants in the high effort condition rated the task as more difficult (*M* = 4.56, *SD* = 1.62) than those in the low effort condition (*M* = 3.70, *SD* = 1.41), *t*_(80)_ = 2.36*, p* = 0.021, Cohen’s *d* = 0.54.

### RewP

**Figure 2:**
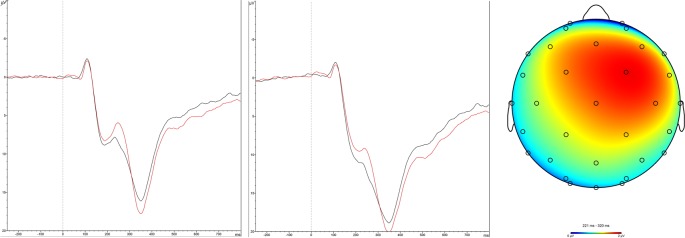
The left-most panel shows the feedback-locked ERPs at Fz for rewards (black) and losses (red). The RewP occurs 220–320 ms after feedback onset (0 on the horizontal axis). The middle panel shows the same event-related potentials (ERP) at Cz. The right-most panel displays the topographic maps of the RewP difference wave, showing that it peaked over frontal-central regions.

The RewP to the reward cue (*M* = 11.94 μV, *SD* = 6.95 μV) was larger than the RewP to the loss cue (*M* = 10.39 μV, *SD* = 6.68 μV), *t*_(81)_ = 3.58, *p* < 0.001 (this is a dependent *t*-test; below, independent *t*-tests are used to compare between-subjects conditions), Cohen’s *d* = 0.39, replicating much past research (see Figure 2). Following recommendations of other ERP/RewP researchers (Luck, 2014; Krigolson, 2018), we created a difference wave by subtracting the RewP in the loss condition from that in the reward condition and used this difference wave as our primary dependent variable.

No difference was found between effort conditions on the RewP difference wave, *t*_(80)_ = 0.09*, p* = 0.930, Cohen’s *d* = 0.02. The amplitude of the RewP was similar in the two conditions (high effort: *M* = 1.53, *SD* = 4.25; low effort *M* = 1.61, *SD* = 3.27).

### Within Condition Correlations

**Figure 3:**
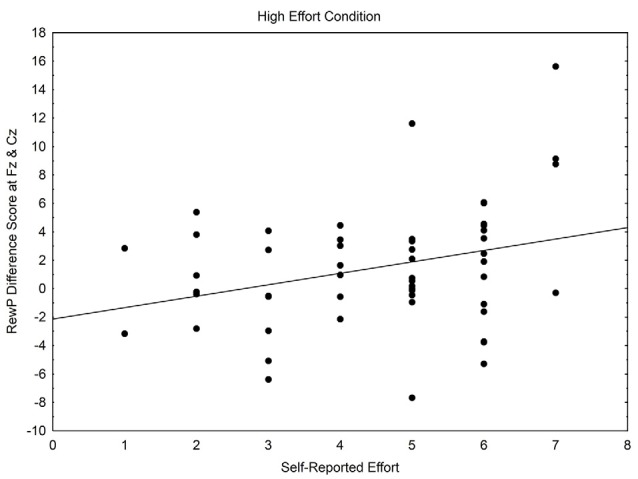
Scatterplot of the relationship between self-reported effort and the reward positivity difference wave, within the high effort condition.

**Figure 4:**
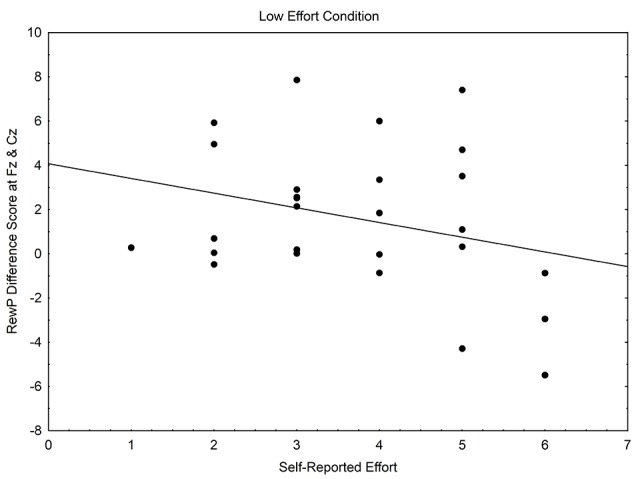
Scatterplot of the relationship between self-reported effort and the reward positivity difference wave, within the low effort condition.

Within-condition correlations, using Pearson’s correlation, were conducted to examine whether perceived effort was related to the RewP. Within the high effort condition, self-reported effort related positively with the RewP difference wave *r*_(53)_ = 0.31, *p* = 0.023, whereas within the low effort condition, a non-significant negative relationship occurred between self-reported effort and the RewP difference wave, *r*_(25)_ = −0.29, *p* = 0.148. See Figures 3, 4.

### Reaction Times and Percent Correct

Participants’ average reaction times to make judgements and the percent of trials correct were calculated. Overall, participants performed well on the task, with the percent of trials correct hovering around 90% and reaction times averaging about 600 ms. However, as expected, the high effort task caused participants to respond more slowly (*M* = 759.39, *SD* = 115.37) and have fewer correct trials (*M* = 88.12, *SD* = 0.056) than the low-effort task (reaction time *M* = 500.24, *SD* = 52.91; percent correct *M* = 92.49, *SD* = 0.048); reaction time *t*_(80)_ = 11.08, *p* < 0.001; percent correct *t*_(80)_ = 3.46, *p* < 0.001.

Based on dissonance theory, we predicted that perceived effort would correlate with RewP, which the above analyses supported. We also analyzed the correlations of perceived effort and RewP with average reaction times and percent correct. Results revealed that within the high-effort condition, RewP did not correlate significantly with percent correct (*r* = −0.16, *p* = 0.24) or reaction time (*r* = −0.01, *p* = 0.93). Perceived effort, however, did correlate significantly with percent correct (*r* = −0.51, *p* < 0.001) but not reaction time (*r* = 0.03, *p* = 0.82).

## Discussion

Results from the present study partially supported the prediction that individuals who believe they have exerted more effort will value the rewards received more. Perceived effort and the RewP were positively correlated within the high effort condition but not within the low effort condition.

Contrary to predictions, the manipulated effort did not exert a significant effect on the RewP. The manipulation of perceived effort may have not been sufficiently strong as evidenced by the results on the self-reported task difficulty manipulation check. That is, the high effort condition reported a mean of 4.56 and the low effort condition reported a mean of 3.70. Although these means differed significantly (*p* = 0.021), the Cohen’s *d* of 0.54 was not large for a manipulation check. A recent meta-analysis of the effect size of manipulation checks (Ejelöv and Luke, 2020) indicated that they tend to be much larger, with a weighted mean effect of Cohen’s *d* = 1.52.

Within the high effort condition, self-reported effort correlated significantly and positively with the RewP, whereas within the low effort condition, no significant correlation occurred between self-report effort and the RewP. This pattern of results may have occurred because participants in the high effort condition were more likely to perceive a contingency between their effort and obtaining the reward, whereas participants in the low effort condition did not. Past dissonance theory research has revealed that effort justification only occurs when individuals perceive a contingency between their effort and obtaining the reward (Gerard and Mathewson, 1966). During the debriefing, three participants reported that they did not believe that their effort would lead to rewards, even though they were not directly asked about this contingency. Thus, perhaps, even more participants doubted a contingency between effort and reward. Moreover, the participants in the low effort condition may have this doubt more than participants in the high effort condition, because participants may have expected to be successful more often in the easier, low effort task. Consequently, the difference in the correlation of effort and RewPs between high and low effort conditions may have been due to the inadvertent result of differences in perceived control between the low and high effort tasks. That is, high effort condition participants perceived more control between their effortful behavior and the rewards than low effort condition participants perceived.

Future research could extend the current research by examining other methods of assessing subjective task difficulty (Hart and Staveland, 1988). Also, future research could extend the present research by assessing ERPs associated with psychological variables that relate to dissonance reduction (e.g., attitudes) in other dissonance-evoking situations (Vanderhaegen et al., 2019), such as the dissonance between positive and negative feelings (Vanderhaegen and Carsten, 2017).

The current study was conducted under the broader perspective of the action-based model of dissonance (Harmon-Jones et al., 2009, 2015a; Harmon-Jones and Harmon-Jones, 2019). This model accepts Festinger (1957) original theory, which proposed that when individuals hold cognitions that conflict with one another they experience a negative affective state (dissonance) which motivates them to alter their cognitions to bring them more into an agreement. The action-based model goes further to propose *why* cognitive conflict is unpleasant. Because cognitions often have action tendencies, conflicting cognitions are likely to motivate incompatible actions, and thus to interfere with effective behavior. The action-based model proposes that reducing the discrepancy between cognitions will, therefore, facilitate effective action, because it brings action tendencies more into alignment. In the case of effort justification, the action-based model proposes that if one has worked hard to obtain a reward, it is generally functional to appreciate that reward and fully utilize its benefits.

## Data Availability Statement

The datasets generated for this study are available on request to the corresponding author.

## Ethics Statement

The studies involving human participants were reviewed and approved by UNSW Human Research Ethics Advisory Panel for Psychology HREAP-C. The patients/participants provided their written informed consent to participate in this study.

## Author Contributions

EH-J and CH-J contributed to the conception and design of the study. DC collected the data with KP’s assistance. DC and KP performed the EEG data processing. DC, KP, CH-J, and EH-J performed the statistical analysis. EH-J and DC wrote the first draft of the manuscript. All authors contributed to manuscript revision, read and approved the submitted version.

## Conflict of Interest

The authors declare that the research was conducted in the absence of any commercial or financial relationships that could be construed as a potential conflict of interest.

## Footnotes

^1^Following the practice blocks of the number judgment task, participants were randomly assigned to one of three between-subjects conditions that differed in the effort and perceived choice. In the high effort/low choice condition, participants read that they had been randomly assigned to engage in the task with blocks that consist of alternating blue and yellow numerals; in the low effort/high choice condition, participants read, “… although it is your choice, we would appreciate it if you would perform the task with blocks that consist of only blue or yellow numerals; and for the high effort/high choice condition they read,” “… although it is your choice, we would appreciate it if you would perform the task with blocks consisting of alternating blue and yellow numerals.” After the number judgment task, the perceived choice was measured by asking participants, “How much choice did you feel you had over engaging in the blocks that consist of only blue or yellow numerals, vs. the block that consists of alternating blue and yellow numerals?” (1 = no choice at all to 7 = a lot of choice). The perceived choice manipulation check question revealed that the choice manipulation was unsuccessful, *F*_(2,78)_ = 2.04, *p* = 0.137. Also, perceived effort correlated positively with the RewP in each of the two high effort conditions (both *r*’s around 0.30), but perceived effort correlated negatively with the RewP in the low effort (i.e., low effort/high choice) condition. As a result, the two high effort conditions are combined.

## References

[B1] Angus D. J. Harmon-Jones E. (2019). The anger incentive delay task: a novel method for studying anger in neuroscience research. Psychophysiology 56:e13290. 10.1111/psyp.13290 30246254

[B2] Angus D. J. Kemkes K. Schutter D. J. Harmon-Jones E. (2015). Anger is associated with reward-related electrocortical activity: evidence from the reward positivity. Psychophysiology 52, 1271–1280. 10.1111/psyp.12460 26084980

[B3] Aronson E. Mills J. (1959). The effect of severity of initiation on liking for a group. J. Abnorm. Soc. Psychol. 59, 177–181. 10.1037/h0047195

[B4] Axsom D. Cooper J. (1985). Cognitive dissonance and psychotherapy: the role of effort justification in inducing weight loss. J. Exp. Soc. Psychol. 21, 149–160. 10.1016/0022-1031(85)90012-5

[B5] Becker M. P. Nitsch A. M. Miltner W. H. Straube T. (2014). A single-trial estimation of the feedback-related negativity and its relation to BOLD responses in a time-estimation task. J. Neurosci. 34, 3005–3012. 10.1523/JNEUROSCI.3684-13.2014 24553940 PMC6608516

[B6] Botvinick M. M. Huffstetler S. McGuire J. T. (2009). Effort discounting in human nucleus accumbens. Cogn. Affect. Behav. Neurosci. 9, 16–27. 10.3758/cabn.9.1.16 19246324 PMC2744387

[B7] Bress J. N. Hajcak G. (2013). Self-report and behavioral measures of reward sensitivity predict the feedback negativity. Psychophysiology 50, 610–616. 10.1111/psyp.12053 23656631

[B8] Carlson J. M. Foti D. Harmon-Jones E. Proudfit G. H. (2015). Midbrain volume predicts fMRI and ERP measures of reward reactivity. Brain Struct. Funct. 220, 1861–1866. 10.1007/s00429-014-0725-9 24549705

[B9] Carlson J. M. Foti D. Mujica-Parodi L. R. Harmon-Jones E. Hajcak G. (2011). Ventral striatal and medial prefrontal BOLD activation is correlated with reward-related electrocortical activity: a combined ERP and fMRI study. NeuroImage 57, 1608–1616. 10.1016/j.neuroimage.2011.05.037 21624476

[B10] Cooper A. J. Duke É. Pickering A. D. Smillie L. D. (2014). Individual differences in reward prediction error: contrasting relations between feedback-related negativity and trait measures of reward sensitivity, impulsivity and extraversion. Front. Hum. Neurosci. 8:248. 10.3389/fnhum.2014.00248 24808845 PMC4009434

[B11] Effort (2009). In MacMillan Dictionary Online. Available online at: https://www.macmillandictionary.com/dictionary/british/effort. Accessed April 20, 2020.

[B12] Ejelöv E. Luke T. J. (2020). “Rarely safe to assume”: evaluating the use and interpretation of manipulation checks in experimental social psychology. J. Exp. Soc. Psychol. 87:103937 10.1016/j.jesp.2019.103937

[B13] Festinger L. (1957). A Theory of Cognitive Dissonance. Stanford, CA: Stanford University Press.

[B14] Foti D. Hajcak G. (2009). Depression and reduced sensitivity to non- rewards versus rewards: evidence from event-related potentials. Biol. Psychol. 81, 1–8. 10.1016/j.biopsycho.2008.12.004 19162124

[B15] Foti D. Hajcak G. (2010). State sadness reduces neural sensitivity to nonrewards versus rewards. Neuroreport 21, 143–147. 10.1097/wnr.0b013e3283356448 20010444

[B300] Gawronski B. Brannon S. M. (2019). “What is cognitive consistency, and why does it matter?” in Cognitive Dissonance: Reexamining a pivotal theory in psychology, 2nd Edn, ed. Harmon-Jones E. (Washington, DC: American Psychological Association), 91–116.

[B16] Gehring W. J. Willoughby A. R. (2002). The medial frontal cortex and the rapid processing of monetary gains and losses. Science 295, 2279–2282. 10.1126/science.1066893 11910116

[B17] Gerard H. B. Mathewson G. C. (1966). The effects of severity of initiation on liking for a group: a replication. J. Exp. Soc. Psychol. 2, 278–287. 10.1016/0022-1031(66)90084-9

[B18] Gratton G. Coles M. G. Donchin E. (1983). A new method for off-line removal of ocular artifact. Electroencephalogr. Clin. Neurophysiol. 55, 468–484. 10.1016/0013-4694(83)90135-9 6187540

[B22] Harmon-Jones E. Amodio D. M. Harmon-Jones C. (2009). “Action-based model of dissonance: a review, integration, and expansion of conceptions of cognitive conflict,” in Advances in Experimental Social Psychology, 41, ed. Zanna M. P. (San Diego, CA: Academic Press), 119–166.

[B19] Harmon-Jones E. Harmon-Jones C. (2019). “Understanding the motivation underlying dissonance effects: the action-based model,” in Cognitive Dissonance: Reexamining A Pivotal Theory in Psychology, 2nd Edition, eds. Harmon-Jones E. (Washington, DC: American Psychological Association), 63–89.

[B23] Harmon-Jones E. Harmon-Jones C. Levy N. (2015a). An action-based model of cognitive dissonance processes. Curr. Dir. Psychol. Sci. 24, 184–189. 10.1177/0963721414566449

[B24] Harmon-Jones E. Price T. F. Harmon-Jones C. (2015b). Supine body posture decreases rationalizations: testing the action-based model of dissonance. J. Exp. Soc. Psychol. 56, 228–234. 10.1016/j.jesp.2014.10.007

[B20] Harmon-Jones E. Mills J. (1999). An introduction to cognitive dissonance theory and an overview of current perspectives on the theory Cognitive Dissonance: Progress on A Pivotal Theory in Social Psychology, eds Harmon-Jones E. Mills J. (Washington, DC: American Psychological Association), 3–21.

[B21] Harmon-Jones E. Mills J. (2019). “An introduction to cognitive dissonance theory and an overview of current perspectives on the theory,” in Cognitive Dissonance: Reexamining A Pivotal Theory in Psychology, ed. Harmon-Jones E. (Washington, DC: American Psychological Association), 3–24.

[B25] Hart S. G. Staveland L. E. (1988). “Development of NASA-TLX (Task Load Index): results of empirical and theoretical research,” in Advances in Psychology Vol. 52, eds Hancock P. A. Meshkati N. (Amsterdam: North-Holland), 139–183.

[B26] Inzlicht M. Shenhav A. Olivola C. Y. (2018). The effort paradox: effort is both costly and valued. Trends Cogn. Sci. 22, 337–349. 10.1016/j.tics.2018.01.007 29477776 PMC6172040

[B27] Krigolson O. E. (2018). Event-related brain potentials and the study of reward processing: methodological considerations. Int. J. Psychophysiol. 132, 175–183. 10.1016/j.ijpsycho.2017.11.007 29154804

[B28] Lange S. Leue A. Beauducel A. (2012). Behavioral approach and reward processing: results on feedback-related negativity and P3 component. Biol. Psychol. 89, 416–425. 10.1016/j.biopsycho.2011.12.004 22178442

[B29] Levy N. Harmon-Jones C. Harmon-Jones E. (2018). Dissonance and discomfort: does a simple cognitive inconsistency evoke a negative affective state? Motiv. Sci. 4, 95–108. 10.1037/mot0000079

[B30] Luck S. J. (2014). An Introduction to the Event-Related Potential Technique. Cambridge, Mass: MIT press.

[B31] Mühlberger C. Angus D. J. Jonas E. Harmon-Jones C. Harmon-Jones E. (2017). Perceived control increases the reward positivity and stimulus preceding negativity. Psychophysiology 54, 310–322. 10.1111/psyp.12786 28118688

[B32] Proudfit G. H. (2015). The reward positivity: from basic research on reward to a biomarker for depression. Psychophysiology 52, 449–459. 10.1111/psyp.12370 25327938

[B33] Sambrook T. D. Goslin J. (2015). A neural reward prediction error revealed by a meta-analysis of ERPs using great grand averages. Psychol. Bull. 141, 213–235. 10.1037/bul0000006 25495239

[B34] Tsypes A. Angus D. J. Martin S. Kemkes K. Harmon-Jones E. (2019). Trait anger and the reward positivity. Pers. Individ. Dif. 144, 24–30. 10.1016/j.paid.2019.02.030

[B35] Vanderhaegen F. Carsten O. (2017). Can dissonance engineering improve risk analysis of human-machine systems? Cogn. Technol. Work 19, 1–12. 10.1007/s10111-017-0405-7

[B36] Vanderhaegen F. Wolff M. Mollard R. (2019). “Synchronization of stimuli with heart rate: a new challenge to control attentional dissonances,” in Automation Challenges of Socio-Technical Systems: Paradoxes and Conflicts, eds Vanderhaegen F. Maaoui C. Sallak M. Berdjag D. (London, UK: Wiley), 3–28.

[B37] Wright R. A. Shaw L. L. Jones C. R. (1990). Task demand cardiovascular response magnitude: further evidence of the mediating role of success importance. J. Pers. Soc. Psychol. 59, 1250–1260. 10.1037/0022-3514.59.6.1250 2283591

[B38] Wright R. A. Tunstall A. M. Williams B. J. Goodwin J. S. Harmon-Jones E. (1995). Social evaluation and cardiovascular response: an active coping approach. J. Pers. Soc. Psychol. 69, 530–543. 10.1037/0022-3514.69.3.530 7562393

[B39] Yaple Z. Shestakova A. Klucharev V. (2018). Feedback-related negativity reflects omission of monetary gains: evidence from ERP gambling study. Neurosci. Lett. 686, 145–149. 10.1016/j.neulet.2018.09.007 30195974

[B40] Yeung N. Sanfey A. G. (2004). Independent coding of reward magnitude and valence in the human brain. J. Neurosci. 24, 6258–6264. 10.1523/JNEUROSCI.4537-03.2004 15254080 PMC6729539

